# ‘The upside-down’ healthcare professional students’ experiences of delirium: an all-Ireland focus group study

**DOI:** 10.1186/s12909-024-06503-x

**Published:** 2024-12-18

**Authors:** Gary Mitchell, Margaret Graham, Jill Murphy, Heather E. Barry, Alice Coffey, Pauline Boland, Tara Anderson, Dympna Tuohy, Matt Birch, Audrey Tierney, Patrick Stark, Arlene McCurtin, James McMahon, Laura Creighton, Elizabeth Henderson, Stephanie Craig, Hannah McConnell, Heather Guttridge, Lana Cook, Emma Cunningham, Geoffrey M. Curran, Christine Brown Wilson

**Affiliations:** 1 School of Nursing and Midwifery, Queen’s University Belfast, Belfast, UK; 2 Department of Nursing and Midwifery, University of Limerick, Limerick, Ireland; 3 School of Pharmacy, Queen’s University Belfast, Belfast, UK; 4 School of Allied Health, University of Limerick, Limerick, Ireland; 5 School of Medicine, Dentistry and Biomedical Sciences, Queen’s University Belfast, Belfast, UK; 6 Center for Implementation Research, University of Arkansas for Medical Sciences, Little Rock, Arkansas, United States

**Keywords:** Delirium, Education, Interprofessional education, Medical student, Pharmacy student, Nursing student, Allied health student, Healthcare

## Abstract

**Background:**

Delirium is a complex neuropsychiatric syndrome characterised by an acute state of confusion, with a substantial impact on medical inpatients. Despite its growing recognition as a global healthcare concern, delirium remains underdiagnosed, partly due to a lack of awareness among healthcare professionals. The aim of this study was to explore how healthcare professional students experience caring for individuals experiencing delirium, the influence of their current pre-registration healthcare education, and importance of interprofessional teamwork in their role.

**Methods:**

This qualitative study used a focus group approach to collect data from 40 healthcare professional students, including nursing, pharmacy, and medical students, across two universities in Ireland. The focus groups explored participants’ experiences of caring for people with delirium, their delirium education, and their collaboration with interdisciplinary teams. The data were analysed using a reflexive thematic analysis approach.

**Results:**

Following thematic analysis, three themes are reported. The first is “The Upside Down,” revealing student perceptions of caring for people with delirium who are facing distressing situations. The second team reported is, “Teamwork Makes the Dream Work,” emphasising the critical role of interprofessional collaboration in delirium management and patient outcomes. Finally, the theme of “A Little Is Not Enough,” highlighted students’ critiques of current delirium education in their pre-registration training. Collectively, these themes illuminate challenges in delirium care, advocate for teamwork in healthcare settings, and call for improvements in educational preparation for future healthcare professionals.

**Conclusions:**

This study contributes to the existing literature by providing insights into the perspectives of healthcare professional students on delirium care. The findings also highlight the challenging nature of caring for individuals with delirium and the need for improved delirium education and interdisciplinary collaboration.

**Supplementary Information:**

The online version contains supplementary material available at 10.1186/s12909-024-06503-x.

## Background

Delirium, also known as an acute confusional state, is a common clinical syndrome characterised by a sudden onset of disturbed consciousness, cognitive function, or perception [1–4]. It typically develops over 1–2 days and has a fluctuating course. Delirium can manifest as either hyperactive, hypoactive, or mixed types, with symptoms varying from heightened arousal and restlessness to withdrawal and lethargy [1–4]. Its prevalence varies across patient populations, with a substantial impact on medical inpatients over the age of 75 years (affecting nearly 20%), over 80% of mechanically ventilated patients, while remaining relatively uncommon in outpatient settings (1–2). Beyond its immediate clinical manifestations, delirium exerts distressing effects that have long-term psychological implications for both patients and their caregivers [3]. The spectrum of cognitive, emotional, and behavioural disruptions associated with delirium encompasses attention, thinking, orientation, memory, emotions, sleep-wake cycle, and behaviour. These disturbances are associated with physical and cognitive impairment or premature mortality [4]. The adverse outcomes of delirium extend to short-term complications such as falls, subsequent sequelae, aspiration pneumonia, and distress, both during the delirious episode and in the post-delirium period [1–4].

Patient experiences of delirium are marked by feelings of powerlessness, anxiety, and a sense of distance from family caregivers and hospital staff [5]. Family caregivers, in the same context, report heightened anxiety and fear, while healthcare professionals often express feeling unprepared, burdened, and stressed when caring for people experiencing delirium. The multifaceted impact of delirium highlights the urgent need for preventive measures [6]. Despite its growing recognition as a global healthcare concern, delirium remains underdiagnosed in primary, secondary, and tertiary care settings [7]. Contributing to this diagnostic gap is a pervasive lack of awareness among healthcare professionals, as evidenced by studies indicating insufficient knowledge among junior doctors regarding the diagnosis and management of delirium [8, 9]. Similarly, nurses have been found to lack clarity on the diagnostic criteria for delirium, often struggling to differentiate between delirium, dementia, and depression [10]. The unpredictable and distressing nature of patients’ disorientation compounds the challenges faced by healthcare professionals, who may lack the requisite knowledge, experience, and resources needed to deliver person-centred care [5].

Early recognition of delirium is paramount as it facilitates the identification of reversible causes and the implementation of interventions tailored to the individual needs of the affected individuals [11–13]. Despite the growing evidence supporting the effectiveness of delirium education in clinical settings, there is a lack of research that exists concerning how delirium education is conveyed and comprehended by pre-registration health profession students encompassing disciplines such as nursing, medicine, pharmacy, and occupational therapy [13]. The limited attention given to the attitudes of healthcare professional students toward delirium, the evaluation of teaching sessions, and the absence of patient and public involvement in delirium education further compound this gap in research [14]. A recent systematic review and meta-analysis has further highlighted the need for increased efforts to enhance the implementation of delirium education and prevention management in clinical practice [15]. The existing evidence also emphasises the urgency of comprehensive research and educational initiatives to address the existing deficiencies in delirium education among health profession students [16–20].

Considering registered professional experience, a recent review highlighted that acute nurses often struggle to prioritise delirium amidst heavy workloads, potentially influenced by ageist perceptions or a lack of support from other professions, leaving the responsibility predominantly on nurses [21]. Conversely, research also demonstrated that early involvement of pharmacists had a significant positive impact on the discharge of patients from the ICU [22]. Further, a recent systematic review found that patients, family members, volunteers, and doctors found comfort in delirium care focused on non-pharmacological interventions [23]. Despite these insights, there can exist a pervasive stigma associated with delirium among healthcare professionals, and particularly those with limited clinical experiences of the condition [24]. While evidence exists on both professional experiences and interventional educational research relating to health professions education, there is however a paucity of empirical research which has explored the experiences of pre-registration healthcare professional students in delirium care. Delirium care for healthcare professional students involves learning to assess, prevent, and manage acute confusion and cognitive changes in patients, ensuring safety, and addressing underlying causes to improve outcomes under the supervision of experienced practitioners. Accordingly, this study sought to explore the following research question; How do healthcare professional students perceive and experience caring for individuals with delirium, and what are their perspectives on pre-registration education and interprofessional teamwork in preparing them for this aspect of care?

## Methods

### Study aim

The aim of this study was to explore how healthcare professional students experience caring for individuals experiencing delirium, the influence of their current pre-registration healthcare education, and importance of interprofessional teamwork in their role.

### Design

This study adopted an interpretive qualitative approach, underpinned by the understanding that the perception of delirium among healthcare professional students is intricately influenced by both individual experiences and the broader social context of their healthcare education [25]. Through the utilisation of focus groups, participants, comprised of healthcare professional students from medicine, nursing, pharmacy and occupational therapy, were encouraged to share their experiences of learning about delirium, working with interdisciplinary colleagues in delirium care and supporting patients with the condition. Employing multiple focus groups allowed for the exploration of commonalities and differences in how healthcare professional students perceive and navigate the challenges associated with delirium [26]. This interpretive qualitative design therefore facilitated holistic understanding of the social and experiential dimensions shaping healthcare professional students’ perspectives on delirium within the context of their education and clinical practice.

### Sample

The study took place in two settings, the University of Limerick (Republic of Ireland) and Queen’s University Belfast (Northern Ireland). The study was conducted using non-probability convenience sampling of second year pre-registration nursing students (*n* = 664), allied health professionals in their final year of study (*n* = 207) and fourth year medical students participating in their geriatric rotation (*n* = 74). These participants were sampled at different stages of their academic programme based on the timing of their clinical placements. To be eligible for this research, students had to have previous experience in providing care to someone living with delirium as part of their clinical placement. In total, 945 healthcare professional students were eligible to participate in this study from both settings.

### Recruitment and consent

Potential participants were contacted by their relevant course lead via an email detailing information and invitation to participate in this study. These gatekeepers were independent and not formally involved in this research. Within the email, students were provided with information sheets and the voluntary nature of involvement was emphasised. A reminder email was circulated twice at two weekly intervals, meaning each eligible participant received a total of three email invitations for this study. Students were required to initiate contact with the research team if interested, placing control in their hands and reducing perceived pressure to participate. Focus group moderators were not involved in any assessment and students were informed that the choice to participate would have no bearing on course progression or module grades assessments. Potential participants were informed that they were also permitted to withdraw from the study at any stage, without giving any reason and information about these processes was provided in the participant information sheet. Students that wished to participate were asked to contact a member of the research team via email to arrange a suitable time and date for their focus group interview. Participants were required to provide written informed consent prior to their focus group session.

### Data collection

Out of the 55 students who initially expressed interest in participating, 46 proceeded to provide written consent. The remaining nine students did not respond to correspondence from the research team after expressing interest. Ultimately, 40 students participated in the study across six focus groups. Focus group one consisted of 11 nursing students, while focus group two comprised 9 nursing students. Focus group three included 3 pharmacy students and focus group four comprised 6 medical students. Additionally, focus group five consisted of 2 occupational therapy students, while focus group six involved 9 nursing students. The focus groups were not mixed, primarily for reasons of convenience, as the sample comprised students from multiple professions across two universities, making it difficult to find mutually suitable times for everyone. Students received no incentive for participation.

Six students who had given consent prior either did not attend on the scheduled day of their interview without giving reason (*n* = 3) or were unable to participate due to illness (*n* = 3). All focus groups took place via Microsoft Teams. This platform was chosen because of convenience as focus groups took place in student’s own time, when they were not timetabled to be on campus. While audio was recorded, no visual data were recorded.

Focus groups were moderated by a combination of the following authors (GM, MG, JM, HB, TA, EH), all of whom have received training in qualitative research and undertaken focus groups prior to this study. The interview guide was developed by the authors in consultation with an expert reference group comprised of clinicians and people with lived experience of delirium. This interview guide focused on three broad areas: (1) experiences of caring for people with delirium, (2) experiences of delirium education from professional course and (3) experiences of working with peers and professionals in the prevention, assessment and management of delirium in healthcare settings. A copy of the consolidated criteria for reporting qualitative studies (COREQ) is available in supplementary file 1.

### Ethical considerations

This study received ethical approval by University Limerick Faculty of Education and Health Sciences (EHSS): (2023_01_02) in March 2023 and Queen’s University Belfast’s Faculty of Medicine, Health and Life Sciences (MHLS) (MHLS23_50) in April 2023. The study took place between April 2023 and October 2023. Written informed consent was obtained from all participants. All methods were performed in accordance with the Declaration of Helsinki. Further, all data and interview transcripts were securely stored and accessible only to the research team. Any direct quotations used in the study were anonymised to prevent the identification of individual participants. These steps were taken to uphold the principles of data privacy and confidentiality in accordance with the Declaration of Helsinki and other relevant ethical guidelines.

### Data analysis

The data were transcribed verbatim and analysed using a reflexive thematic analysis approach, as outlined by Braun and Clarke [27]. The analysis involved a recursive, six-stage process. First, the research team (GM, MG, JM, HB) familiarised themselves with the data through repeated reading and by writing initial reflective notes. Second, the team generated initial codes, paying close attention to patterns of shared meaning across the dataset. Third, the team collaboratively generated initial themes, discussing and debating how different codes could be combined to capture broader conceptual ideas. Fourth, the themes were reviewed and refined, with the team critically examining whether the themes accurately reflected the coded data. Fifth, the themes were further defined and named, with the team articulating the ‘essence’ of each theme. Finally, the analysis was compiled into a coherent written report. Importantly, the team engaged in reflexive discussions throughout the analytic process, reflecting on how their own backgrounds, assumptions and perspectives might be shaping the interpretation of the data. The reflexive thematic analysis took place through an iterative process where the research team continuously engaged in self-reflection and discussion, critically examining their own influence on the interpretation of the data. The reason for using this approach was to ensure that the analysis remained deeply rooted in the participants’ experiences, thereby minimising the impact of the researchers’ biases and assumptions on the findings [27].

### Trustworthiness

In enhancing the reliability of this data, this research adhered to the four criteria of credibility, transferability, dependability, and confirmability [28]. Participants were actively engaged in the process, given the opportunity to review both the raw and themed data, fostering transparency and inclusivity. Rigorous record-keeping practices were consistently implemented and maintained throughout the entire research project. To ensure the integrity of our approach, regular team meetings were conducted with all co-authors, providing a platform for comprehensive discussions and serving as a mechanism to establish a clear audit trail.

## Results

40 students participated across six focus groups. These focus groups lasted between 30 and 60 min. Table 1 provides an overview of the demographics of the participants.

**Table 1:** Participant Demographic Details

**Gender**	Male	6
	Female	34
**Age**	18–25	27
	26–35	8
	36–45	2
	46–55	3
**Health Profession Discipline**	Medicine	6
	Pharmacy	3
	Nursing	29
	Occupational Therapy	2

Following thematic analysis, three discernible themes became apparent. These were, *“the upside-down”*, whereby participants discussed their perceptions of delirium, “*teamwork makes the dream work*”, when participants discussed the extent to which they worked with peers or professional colleagues to provide care to people with delirium and “*a little is not enough*” which described the perceived usefulness of delirium education within their programme alongside recommendations for improvement. Supplementary file 2 provides a coding example from this study.

### Theme one: “The upside-down”

Students from medicine, nursing, pharmacy and occupational therapy participating in the focus groups often recounted challenging experiences when faced with people who were experiencing possible symptoms of delirium. One student described their first experience of delirium as follows, *“it was a bit frightening to be honest…confusion*,* paranoia*,* hallucinations – you name it*,* it was all there*,* and it was bloody difficult.”* [FG1, Nursing Student 8]. Another student with placement experience on an older people’s hospital ward stated, *“I struggled with it [delirium care] …well*,* I’d ought to say struggle…you know present tense [laughs].”* [FG4, Medical Student 3]. When describing these challenging experiences, student participants often spoke in the context of caring for a person with delirium, for example difficulties in reducing patient distress, difficulties in reorientating patients to the correct time or place and overcoming patient suspiciousness. An example is noted in the following excerpt, “*I think the most challenging thing was building a rapport…when patients with delirium don’t trust you or what*,* it means they aren’t going to take their medications or their drips to get better and that is a pickle”.* [FG2, Nursing Student 3].

The emotional impact of caring for people with delirium was a prevalent topic of conversation amongst many student participants. Some participants stated that they felt sad when reflecting on the care they were able to provide people with delirium, while others said they thought about the care of these patients long after leaving placement as noted here, “*the patient I am referring to [with delirium]*,* I think about her quite often. I think about the things I could have done to help her more”* [FG2, Nursing Student 5]. When considering the trajectory of delirium, healthcare professional students reflected upon the entire patient journey, from rapid onset, to fluctuating behaviour, to eventual recovery. The journey into delirium was noted as being particularly challenging for healthcare professional students, with expressions of uncertainty about what to expect during care interactions. One nursing student shared, *“It was like delirium had its own script; you never knew what version of the person you were going to meet on any given day.”* [FG1, Nursing Student 2]. Another occupational therapy student stated, *“One day I was there with this man*,* he was lovely. I went back to the ward in a few days later he was completely different*,* he was aggressive and agitated*,* later I learnt he had delirium.”* [FG5, Occupational Therapy Student 2]. While a medical student stated, *“I remember there were three or four of us [medical students] on the ward and we were doing like a debrief session with one of the consultants and I think one of the things that stuck with me anyway*,* was the sheer fluctuations from being lucid to manic and back again in a few hours”*. [FG4, Medical Student 1].

Contrary to their initial expectations, some students witnessed positive outcomes in delirium care, challenging preconceptions about delirium’s lasting impact on memory. Patients emerged from delirium, surprising students with their ability to recall unsettling experiences, adding a layer of complexity to the care. *“Yes*,* the key difference…to say something like dementia that maybe seems similar [to the presentation of delirium in patients]*,* was that after treatment*,* the lady I looked after had total recall*,* remembered everything about their experience. I thought that must have been tough [for the patient]”.* [FG1, Nursing Student 10]. Overall, healthcare professional students articulated delirium experiences that they often perceived to be strange or unusual. This was evidenced in the reflection of a nursing student who directly referenced the Netflix Television Series ‘Stranger Things’. During focus group two the participant remarked, *“To me*,* delirium is like the upside-down world from Stranger Things…patients with delirium are seeing and experiencing a sort of distorted version of our reality”.* [FG2, Nursing Student 6].

Overall, these varied perspectives provide valuable insights into the multifaceted nature of delirium experiences from participants. Overall, participants spoke most about caring for people with hyperactive delirium during their clinical placement at a hospital or nursing home settings. While some participants acknowledged hypoactive delirium, for example naming symptoms like drowsiness, lethargy and sluggishness, the majority focused on their experiences of hyperactive symptoms.

### Theme two: “Teamwork makes the dream work”

The second theme extracted from the data reflected the collaborative nature of delirium care, as described by the healthcare professional students who participated in the study. The theme emphasises the importance of interprofessional teamwork in providing care to individuals experiencing delirium. The students discussed their experiences of working with peers and professional colleagues in the prevention, assessment, and management of delirium in healthcare settings. Together, they shared insights into the challenges and rewards of collaborating with interdisciplinary professionals to support patients with delirium. With regards to patient assessment, the participants discussed the importance of the teamwork in obtaining a diagnosis as noted here, *“Think delirium*,* that is what I always say. If you suspect it*,* all you need is a quick 4AT [Validated delirium assessment tool] and you’re off to the races. Once you get the confirmation*,* then it is over to your doctors or whatever to find out the underlying cause.”* [FG2, Nursing Student 5]. To further compliment this, medical students highlighted that they often relied on their nursing colleagues to undertake delirium screening, and this was invaluable to their role as evidenced in the following two quotes. Firstly, *“They [Nurses] know the patient best in most cases really. So*,* it is actually pretty normal that they [Nurses] will be the ones to alert us or pick it up first and once they bring it to our attention*,* we can kind of just act on it…get it sorted”.* [FG4, Medical Student 4]. Secondly, *“Without a doubt*,* it’s the nurses. We rely on them – particularly for patients who are hypo (Hypoactive Delirium)*,* we might not spot them on the round [Ward Round]”.* [FG4, Medical Student 1].

Of equal importance to the assessment of delirium, participants also spoke about the importance of teamwork in the management and prevention of the condition. Focus group participants consistently asserted the importance of teamwork with each interdisciplinary professional having a role to play in supporting people experiencing delirium. Pharmacy students illuminated how their role often involved medication management after diagnosis as reported here whereby antibiotic therapy, reduction in polypharmacy or analgesia could inform treatment plans for delirium, *“What comes to mind most is in cases of UTI [urinary tract infection]*,* this whole behaviour changes so quickly [because of delirium caused by UTI]. Also*,* not just UTIs*,* but when like the medicines change*,* conditions deteriorate in general or at end of life”.* [FG3, Pharmacy Student 2]. Another aspect that was considered was the role of physiotherapy, *“Mobility is a big factor [in delirium management]. Get them moving [the patient] or if they have had a fall or break [fracture]*,* get physio input*,* and get mobilising as soon as possible”.* [FG1, Nursing Student 1]. While participants highlighted several professional colleagues, nursing students also mentioned the importance of activity coordinators and healthcare workers, particularly within the care home setting. *“What about the activity coordinator from the nursing home where I work? She maybe wouldn’t know she is treating possible or potential delirium to be fair*,* but by keeping resident minds active with puzzles or books or reminiscence that would improve the delirium I think.”* [FG2, Nursing Student 4]. Participants consistently highlighted how everyone had a role to play in delirium management in both hospital and care home settings. This was succinctly summarised by a medical student who stated, *“We all bring our own specialties and strengths to the table*,* after all*,* teamwork makes the dream work”.* [FG4, Medical Student 3].

Overall, the theme summarises the experiences and perceptions of healthcare professional students regarding the collaborative nature of delirium care. It emphasises the pivotal role of interprofessional teamwork in ensuring holistic and effective support for individuals affected by delirium. Notably, there were seldom instances where participants discussed the challenges of interdisciplinary approaches to delirium care, as they instead chose to focus on the facilitators to good practice.

### Theme three: “A little is not enough”

Theme three moved away from discussions about personal experiences of delirium (theme one) and discussions about interdisciplinary teamwork in delirium care (theme two). Instead, this theme focused on student perceptions on the education they had received about delirium prior to providing direct patient care on clinical placements and how these might influence student experience. Prior to supporting people with delirium, participants highlighted differences in the provision of their professional programme. Medical students articulated clear learning objectives aligned to delirium in their programme but highlighted that the detail of delirium education was often different because this was influenced by their practice placement and their supervising doctor, *“Yeah*,* we definitely do quite a bit on delirium and yes*,* I would also say that is explicitly covered in the degree [programme]. Maybe the only thing to say is that the level of detail each medical student gets is pretty dependent on where you are working. So*,* for example*,* I am in geriatrics now and because delirium happens a lot there*,* I’ve had a bit more training and education from the consultants that work there”.* [FG4, Medical Student 2]. This was contrasted to nursing and pharmacy students that participated in the focus groups, with both groups acknowledging that there was less clear delirium education within their curricula. One participant noted, *“I don’t think delirium is that well covered to be honest”.* [FG1, Nursing Student 3]. Similarly, a pharmacy student stated, *“I think probably everyone has a vague awareness of what it is*,* but I don’t remember being told*,* alright*,* this is what delirium is…so no*,* I’d say very little exposure over the four years [of the course]”.* [FG3, Pharmacy Student 2]. Finally, the following quotation appeared to summarise much of the narrative around delirium education in nursing and pharmacy programmes, *“It’s [delirium] not really covered across the three years. I mean there was a podcast and lesson in year one I am pretty sure*,* but that was not really reinforced or developed after. It was a little [delirium education] I guess…and a little is not enough”.* [FG2, Nursing Student 1].

Participants collectively moved discussions around their prior delirium learning towards future recommendations for delirium education within their programme. Participants outlined several key elements they deem essential when designing education about delirium care. The key recommendation that emerged from the focus groups was the importance of framing delirium education as interdisciplinary. As highlighted in theme two, participants clearly indicated that working together was an effective part of delirium care. Furthermore, focus group participants also expressed a strong preference towards digital education as a mode for delirium education. One nursing student stated, *“I like to see visual elements to learn and find it easier when I see a picture”.* [FG5, Nursing Student 3]. While a medical student added, *“I like the idea of a cyber resource or online platform or something that just takes through everything and tells you*,* like*,* nurses do this in assessment and say us medics do this and wait there is pharmacy and they come in here and discharge planning*,* well then boom*,* you’ve got the occupational therapists and whatever…do you know what I mean?”* [FG4, Medical Student 4]. While there was mostly consensus for this type of digital e-resource, some participants disagreed about the level of detail required. While medical students consistently recommended a high level of information, pharmacy and nursing students displayed variability in the level of information they required. For example, one pharmacy student stated, *“I think not having too much information on it. The BNF [British National Formulary Application] works well because it has those four sections at the bottom*,* and it is easy enough to navigate. In other apps*,* sometimes I am like*,* I don’t know where to look because there is so much on it”.* [FG3, Pharmacy Student 2]. Similarly, a nursing student illustrated the importance of considering practicalities of digital education, *“I need to be able to see the screen on my phone screen*,* if I am using a tablet*,* friendly for both”*. [FG5, Nursing Student 9].

Theme three diverged from shared interprofessional experiences of delirium and discussions on interdisciplinary teamwork to focus on student perceptions of the delirium education they received prior to engaging in direct patient care during clinical placements and their recommendations for future education about delirium. As noted, medical students emphasised clear learning objectives aligned with delirium in their curriculum but noted variations in education depth depending on their practice placement. Conversely, nursing and pharmacy students indicated less explicit delirium education in their programmes. Suggestions for improvement included framing delirium education as interdisciplinary and utilising digital resources, although opinions varied on the desired level of detail. Nursing and pharmacy students emphasised practical considerations for digital education accessibility, while medical students appeared to recommend a higher level of detail was required.

## Deviant case analysis

While the results predominantly reflect common themes regarding healthcare professional students’ experiences with delirium care, it is important to acknowledge potential deviant cases that were less frequently reported. Reporting these deviant cases is essential to ensure a comprehensive analysis and to capture the full spectrum of experiences. Interestingly, a few participants did mention that family members played a crucial role in understanding the person behind the delirium and aiding in the assessment due to their pre-illness knowledge of the patient. Despite this, only a few students highlighted this positive aspect, whereas a broader expectation would suggest more students might have reported similar experiences. Furthermore, other examples of deviant cases in the study included differing opinions on the effectiveness of delirium education and teamwork. While most participants emphasised the importance of interprofessional collaboration, a few had little insight into the role or importance of teamwork in delirium. Additionally, while many students discussed the importance of developing interprofessional digital delirium education, there was a small minority of participants that suggested that traditional face-to-face lectures would be better. Regarding education, there was also a small number of students that believed that delirium education might be best taught within a clinical setting as opposed to a higher education institution.

## Discussion

Participants articulated the multifaceted nature of delirium, emphasising its sudden onset and fluctuating symptoms, which complicate both patient assessment and management. This aligns with existing literature that highlights the need for heightened awareness and training among healthcare professionals to recognise delirium promptly and accurately [1–4]. The experiences shared by healthcare professional students in the study regarding delirium care also concord with existing literature that emphasises the challenging and distressing nature of caring for individuals with delirium [29–34]. Previous research has also highlighted healthcare providers’ struggles in supporting people with delirium in care, particularly in reorienting them, reducing patient distress, and building rapport [35–38]. The emotional impact on student healthcare professionals, as seen in reflections on the unpredictability of delirium care interactions and the lasting effects on patients and caregivers, resonates with findings from other studies that have focused on professional audiences. On the other hand, there appears to be a paucity of empirical findings that have highlighted successful or rewarding experiences of delirium care, and these were noted in the present study. This finding offers contrast with empirical findings and evidence synthesis papers which often report on the negative personal experiences associated with caring for people with delirium [29, 38].

Within this study, participants highlighted the importance of interprofessional collaboration in managing delirium effectively. They shared detailed experiences, noting how coordination between nurses, physicians, pharmacists, and other allied health professionals is crucial in assessing, managing and preventing delirium. Their accounts emphasised how teamwork among different healthcare disciplines can enhance patient outcomes by fostering comprehensive care approaches that address both medical and psychosocial needs. Participants specifically mentioned the value of regular communication and shared decision-making in ensuring that all aspects of a patient’s health are considered, from physical symptoms to emotional well-being. The emphasis placed by participants on the importance of teamwork in delirium care is also consistent with previous literature advocating for interdisciplinary collaboration in managing people who are experiencing delirium [39–42]. Studies have shown that collaborative approaches involving various healthcare professionals lead to improved patient outcomes, enhanced communication, and more effective care delivery for individuals with delirium [20, 43–47]. This includes reducing the length of hospital stays, minimising medication errors, and ensuring continuous monitoring of patients’ mental status. The findings from this study align with existing research highlighting the significance of teamwork in providing holistic care to patients experiencing delirium. Despite this finding, there is a lack of research that has explicitly explored teamwork in the context of healthcare professional student practice or education [32]. This gap suggests a need for further investigation into how interprofessional collaboration is taught and reinforced during healthcare training, as this may play a critical role in preparing future professionals for managing complex conditions like delirium.

Participants’ recommendations for enhancing delirium education within their programmes, particularly their preference for digital education resources, reflect a growing trend towards technology-enhanced learning in healthcare education. While digital education resources offer benefits such as providing visual learning aids, interactive platforms, and accessibility across various devices, challenges exist regarding the level of detail required to effectively convey complex information, as well as accommodating individual preferences for information depth and learning styles [48–50]. Within higher education in delirium, there have been several digital interventions that have shown promising evidence with regards to enhancing student knowledge, self-efficacy and practice using methods such as serious gaming, e-learning, virtual reality and podcasting [16, 51–57]. Therefore, participant recommendations regarding digital delirium education are likely to be feasible and have the potential to be effective. One aspect that is less common in the literature, pertains to interdisciplinary education about delirium for healthcare professional students with most interventions focusing on either medical or nursing students (16, 51–52, 54, 55, 56, 57). It appears as only one empirical study, focused on a digital serious game, has sampled both nursing and medical students [53].

In contrast to the preference for digital education resources, some literature suggests that face-to-face or traditional educational methods may be more effective in certain contexts. This is because face-to-face interactions can provide personalised learning experiences, immediate feedback, and opportunities for hands-on practice that may be lacking in purely digital formats. However, the evidence base provides mixed results with literature suggesting that digital education has the potential to be more effective or less effective than traditional modes of teaching based on a variety of factors such as the characteristics of the learners, the subject matter being taught, and the specific learning objectives [58–60]. Factors such as the learners’ digital literacy, access to technology, and individual learning preferences can influence the effectiveness of digital education [58–60]. Additionally, the nature of the content itself, including its complexity and the need for interactive engagement, can impact the suitability of digital versus traditional methods. Moreover, the context in which learning takes place, such as the availability of resources, time constraints, and institutional support for technology integration, also plays a significant role in determining the effectiveness of digital education. Therefore, while some studies advocate for the superiority of face-to-face teaching, others argue that digital education offers unique advantages, such as flexibility, scalability, and accessibility, particularly in reaching diverse learner populations and addressing specific learning needs [58–73].

## Strengths, limitations and recommendations

Strengths of this study include its novel approach in exploring the experiences of healthcare professional students in delirium care, filling a significant gap in the literature. The use of focus groups allowed for rich data collection, capturing diverse perspectives across different disciplines. The sample also represented healthcare professional student voices across two different institutions in the country of Ireland and three professional subjects (medicine, nursing and pharmacy). However, there are limitations to consider. The study’s reliance on convenience sampling may introduce selection bias, potentially limiting the transferability of findings. Additionally, the focus on healthcare professional students at specific stages of their academic programmes may not fully capture the breadth of experiences across different levels of training. A further limitation of this study was the wide variation in focus group sizes, ranging from 3 to 11 participants. This inconsistency may have affected the depth and breadth of discussions across groups, potentially impacting the quality and comparability of data collected. Smaller groups might have generated less diverse perspectives, while larger groups could have been more challenging to moderate effectively.

Future research should consider employing more diverse and representative sampling methods to ensure broader inclusivity of healthcare professional students and include disciplines from other fields of allied health. Longitudinal studies examining students’ experiences and learning outcomes over time could provide deeper insights into their experiences in delirium care and education. Furthermore, incorporating mixed-methods approaches could offer complementary quantitative data to enrich qualitative findings. Regarding delirium education, participants have clearly articulated their desire for increased delirium education in their programme and in particular, development of digital delirium education that is focused on interdisciplinary care.

## Conclusion

This study adds to the existing literature by emphasising the challenging nature of caring for individuals with delirium and the crucial role of teamwork in delivering holistic care from the perspective of healthcare professional students. While previous research has primarily focused on the experiences of registered professionals, this study is among the first to explore the collective perspectives of interdisciplinary healthcare professional students. The findings demonstrate that interprofessional teamwork and supportive education can significantly aid students in their practice. Additionally, the study offers recommendations for integrating digital education resources to enhance delirium education for healthcare professional students.

This study further contributes to the existing literature by providing valuable insights into the perspectives of healthcare professional students on delirium care. The findings illustrate the complex nature of caring for people with delirium and highlight the need for improvements in delirium education and interdisciplinary collaboration. Specifically, students expressed the need for enhanced practical training, with more hands-on experience in delirium management, as indicated by the theme “A Little Is Not Enough.” The theme “Teamwork Makes the Dream Work” also emphasised the importance of interprofessional education to improve care outcomes. Furthermore, the “Upside Down” theme revealed a gap in addressing the emotional and psychological aspects of delirium care, and students noted a disconnect between theory and practice, calling for more integrated educational approaches. Lastly, students critiqued the limited focus on delirium in current curricula, advocating for more comprehensive coverage of the topic. Implementing these educational improvements could better prepare future healthcare professionals to provide compassionate and effective care for patients experiencing delirium.

## Electronic Supplementary Material

Below is the link to the electronic supplementary material.

Supplementary Material 1

Supplementary Material 2

## Data Availability

The datasets used and/or analysed during the current study are available from the corresponding author on reasonable request.
