# Supplementary material for: ‘The upside-down’ healthcare professional students’ experiences of delirium: an all-Ireland focus group study
Source: BMC Med Educ. 2024 Dec 18;24:1470. doi: 10.1186/s12909-024-06503-x (PMC11654305; doi:10.1186/s12909-024-06503-x)
Supplement: Supplementary file 1 — Supplementary Material 1 [file 12909_2024_6503_MOESM1_ESM.docx]

**Supplementary File 1**

Consolidated criteria for reporting qualitative studies (COREQ): 32-item checklist.

| **No** | **Item** | **Guide questions/description** |
| --- | --- | --- |
| **Domain 1: Research team and reflexivity** |  |  |
| Personal Characteristics |  |  |
| 1. | Interviewer/facilitator | All interviews were facilitated by two of the following authors: Dr Gary Mitchell (GM), Dr Margaret Graham (MG), Dr Jill Murphy (JM), Dr Heather Barry (HB) or Miss Tara Anderson (TA). |
| 2. | Credentials | GM, MG, JM, HB and TA have expertise in qualitative research methods and qualitative data collection (including semi-structured interviewing and focus-group interviewing). |
| 3. | Occupation | GM is a Reader at Queen’s University Belfast in Northern Ireland. MG & JM are Lecturers at University of Limerick in Ireland. HB is a Lecturer and TA is a Research Assistant at Queen’s University Belfast in Northern Ireland. |
| 4. | Gender | GM is male. MG, JM, HB, TA are female. |
| 5. | Experience and training | GM, MG & JM are registered nurses with considerable experience in higher education and care of older people living with delirium. HB is a pharmacist with considerable experience in higher education and care of older people. TA is a research assistant with a background in psychology and care of older people. |
| Relationship with participants |  |  |
| 6. | Relationship established | GM, MG & JM teach on the Undergraduate Nursing Programmes where students were nursing students were recruited. HB teaches on the undergraduate Pharmacy Programme where pharmacy students were recruited. TA had no previous relationship with participants. |
| 7. | Participant knowledge of the interviewer | Participants would have been aware of at least one person who was conducting the focus groups as these were detailed in the information sheet and consent forms. |
| 8. | Interviewer characteristics | GM, MG, JM, HB are registered healthcare professionals with expertise in the topic area. TA has a strong awareness in the context (e.g., interdisciplinary delirium education and qualitative research). |
| **Domain 2: study design** |  |  |
| Theoretical framework |  |  |
| 9. | Methodological orientation and Theory | Interpretative qualitative approach using six focus groups. |
| Participant selection |  |  |
| 10. | Sampling | Convenience sampling of 945 health professional students undertaking their nursing, medicine, pharmacy or occupational therapy degree at Queen’s University Belfast or University of Limerick. |
| 11. | Method of approach | Participants were approached by a gatekeeper (Director of Education or equivalent) that was not associated with the study. |
| 12. | Sample size | 40 participants. |
| 13. | Non-participation | Participants were reminded that participating in this research would not affect their course grade. This was noted in the information sheet and within the consent form. |
| Setting |  |  |
| 14. | Setting of data collection | All six focus groups took place online. All data was collected via online meetings (MS Teams). Data collection took place during the student’s own time. |
| 15. | Presence of non-participants | There were no non-participants present during the focus group interviews. |
| 16. | Description of sample | Participants were undertaking pre-registration training in either nursing, medicine, pharmacy or occupational therapy at either Queen’s University Belfast (Northern Ireland) or University of Limerick (Republic of Ireland). |
| Data collection |  |  |
| 17. | Interview guide | The interview guide was designed by the authors with input from an expert reference group. |
| 18. | Repeat interviews | No repeated interviews were carried out. |
| 19. | Audio/visual recording | All focus group data was audio-recorded. Focus groups were not visually recorded. |
| 20. | Field notes | No field notes were collected during focus group interviews. |
| 21. | Duration | Focus group interviews lasted approximately 40 minutes on average (range: 26 minutes to 57 minutes). |
| 22. | Data saturation | Data saturation was achieved in this study with the participation of 40 healthcare professional students in 6 focus groups, wherein recurring themes and perspectives relevant to the study question were consistently observed, indicating comprehensive exploration of the topic. |
| 23. | Transcripts returned | All participants had the option of reviewing their focus group transcript as noted in the information sheet, consent form and at the conclusion of the focus group interview. No participant requested this. |
| **Domain 3: analysis and findings** |  |  |
| Data analysis |  |  |
| 24. | Number of data coders | Qualitative data analysis, using thematic analysis (Braun and Clarke), was carried out by GM, MG, JM, HB, TA and CBW). |
| 25. | Description of the coding tree | All authors actively participated in the design, data collection, analysis, and interpretation of the study, ensuring comprehensive collaboration and shared contributions to the research process. |
| 26. | Derivation of themes | The authors used thematic analysis to analyse themes. This was led by GM, MG, JM, HB, TA & CBW initially and involved all team members in the advanced stages. |
| 27. | Software | The authors did not use software to generate their themes. |
| 28. | Participant checking | No member checking was carried out, however participants were asked if they wished to review the transcript from their focus group. |
| Reporting |  |  |
| 29. | Quotations presented | Direct quotations were presented from participants while confidentiality was maintained. |
| 30. | Data and findings consistent | The authors have ensured there was concordance between the data and findings that are presented. All authors were involved. |
| 31. | Clarity of major themes | The authors have reported all major themes as identified in this research. |
| 32. | Clarity of minor themes | The authors were not required to report on any minor themes or deviant cases within their presentation of results as these did not emerge. |
