# Supplementary material for: ‘The upside-down’ healthcare professional students’ experiences of delirium: an all-Ireland focus group study
Source: BMC Med Educ. 2024 Dec 18;24:1470. doi: 10.1186/s12909-024-06503-x (PMC11654305; doi:10.1186/s12909-024-06503-x)
Supplement: Supplementary file 2 — Supplementary Material 2 [file 12909_2024_6503_MOESM2_ESM.docx]

**Supplementary File 2**: **Coding Example**

**Basic Coding:**

1. Delirium symptoms impact student perceptions
2. Initial fear and challenges described by students
3. Emotional impact on students caring for delirious patients
4. Uncertainty and unpredictability of delirium experiences
5. Positive outcomes observed in delirium care
6. Reference to "Stranger Things" metaphor
7. Hyperactive delirium within clinical settings
8. Interprofessional teamwork highlighted in delirium care
9. Role of nursing in delirium assessment
10. Pharmacy role in medication management for delirium
11. Importance of physiotherapy in delirium management
12. Activity coordinators' role in care home settings
13. Variability in delirium education across healthcare programs
14. Medical students' detailed curriculum on delirium
15. Nursing and pharmacy students' lack of delirium education
16. Recommendations for multidisciplinary delirium education
17. Preference for digital learning resources
18. Visual elements preferred for learning about delirium
19. Medical students advocate for comprehensive e-resources
20. Pharmacy students' preference for concise digital resources
21. Nursing students' need for mobile-friendly digital education
22. Challenges in navigating information-rich digital resources
23. Impact of practice placements on delirium education depth
24. Emotional reflections on patient care beyond clinical settings
25. Student perceptions of patients' recovery from delirium
26. Fluctuating nature of delirium symptoms discussed
27. Psychological and behavioural aspects of delirium
28. Patient-centred care approaches in delirium management
29. Reflections on improving patient rapport in delirium care
30. Impact of delirium on memory and patient recall

**Intermediate Coding:**

**Theme One: “Stranger Things and the Upside Down”**

- Emotional impact and challenges in delirium care
- Metaphorical reference to "Stranger Things" in delirium experiences
- Student reflections on patients' unpredictable behaviours
- Fear and uncertainty in caring for people with delirium

**Theme Two: “Teamwork Makes the Dream Work”**

- Importance of interprofessional collaboration in delirium care
- Nursing role in delirium assessment and patient care
- Pharmacy's role in medication management for delirium
- Physiotherapy's contribution to mobility in delirium management

**Theme Three: “A Little Is Not Enough”**

- Variability in delirium education across healthcare programs
- Medical students' detailed curriculum on delirium
- Nursing and pharmacy students' lack of delirium education
- Recommendations for multidisciplinary and digital delirium education
